# Predictors of high-grade atherosclerotic renal artery stenosis in patients with CKD

**DOI:** 10.1097/MD.0000000000041007

**Published:** 2024-12-27

**Authors:** Jun Ouyang, Kequan Chen, Hui Wang, Jiangnan Huang

**Affiliations:** a Department of Cardiology, The First Affiliated Hospital of Guangxi Medical University, Nanning, China; b School of Pharmacy, Guangxi Medical University, Nanning, China.

**Keywords:** CKD, cystatin C, eGFR, high-grade ARAS, LMR

## Abstract

This study aims to explore predictors of high-grade atherosclerotic renal artery stenosis (ARAS) in patients with chronic kidney disease (CKD). This was a retrospective study, and univariate analysis such as independent-sample t test or nonparametric test where appropriate was used to explore variables with significant difference between patients with high-grade ARAS and patients with low-grade ARAS. Then, multivariate logistic regression and receiver operating characteristic curve (ROC) analysis were performed for further research. In univariate analysis, we found that there was a significant difference in smoking history, estimated glomerular filtration rate (eGFR), cystatin C, fasting blood glucose and lymphocyte-to-monocyte ratio (LMR) between the 2 groups. Multivariate logistic regression analysis showed that eGFR (OR = 0.979, 95% CI: 0.962–0.996, *P* = .017), cystatin C (OR = 2.123, 95% CI: 1.118–4.030, *P* = .021) and LMR (OR = 0.639, 95% CI: 0.421–0.969, *P* = .035) were still associated with high-grade ARAS in patients with CKD. ROC analysis showed that eGFR (AUC: 0.681; sensitivity: 64.1%, specificity: 65.1%), cystatin C (AUC: 0.658; sensitivity: 74.6%, specificity: 53.85%) and LMR (AUC: 0.650; sensitivity: 66.70%, specificity: 62.00%). In patients with CKD, eGFR, and cystatin C and LMR were predictive parameters of high-grade ARAS, and among them, eGFR and LMR held the greatest predictive value for high-grade ARAS in patients with CKD.

## 1. Introduction

Renal artery stenosis (RAS), especially high-grade RAS, is a main contributor to hypertension and chronic kidney disease (CKD).^[1–3]^ Among all factors leading to RAS, atherosclerotic RAS (ARAS) is the most common. In the current study, high-grade RAS was recognized as more than 50% stenosis according to a previous study.^[4]^ To date, the incidence and prevalence of RAS are unknown because the clinical manifestations of RAS are insidious and have no specificity. Patients with coronary artery disease (CAD) or other atherosclerotic peripheral vascular disease are considered to be at greater risk for RAS.^[5,6]^ The most common presentations are renovascular hypertension and abnormal renal function. At present, the treatment strategies for RAS (drug therapy alone or drug plus renal artery stent implantation) have been controversial in the medical field. Recently, evidence from 3 large randomized controlled trials (RCTs), namely, STAR,^[7]^ ASTRAL,^[8]^ and CORAL,^[9]^ has refuted the superiority of percutaneous renal artery revascularization compared to medical treatment. However, the study designs of these RCTs have some limitations, such as lack of sufficient patients with high-grade RAS or the recruitment of patients with severe chronic renal function damage. Therefore, at present, it is more important for clinicians to identify selected patients who may benefit from revascularization.

People with high-grade RAS have been given more attention than those with low-grade stenosis because people with high-grade stenosis may be more likely to benefit from renal artery revascularization. In 2021, the kidney disease: improving global outcomes (KDIGO) organization issued a consensus on indications and nonindications for renal artery revascularization in atherosclerotic renovascular disease.^[10]^ In general, high-risk patients, such as those with high-grade RAS, presenting with acute cardiopulmonary or acute renal insufficiency or having a history of renal transplantation, were definite indications. Consistent with KDIGO guideline, in 2022, a single-center retrospective cohort study, a prospective 2-center cohort study and a case report all reported that successful renal revascularization was conducive to restore renal function and reduce the coincidence of hospital admissions for heart failure syndromes for high-grade RAS patients with above acute presentations.^[11–13]^ Patients with high-grade RAS were more prone to life-threatening complications than those with low-grade RAS. Therefore, clinical practitioners should recognize in time those patients with high-grade RAS who temporarily have no severe complications to determine corresponding therapy according to individual situations. Clinically, however, it is difficult to detect RAS, even in patients with high-grade RAS, unless patients present with severe acute heart failure, renal insufficiency or refractory hypertension. The existence of high-grade RAS may further damage kidney structure and function, leading to CKD progression by inflammation, hypoperfusion and oxidative stress mechanisms.^[14,15]^ Therefore, we should pay attention to high-grade RAS in patients with CKD.

ARAS accounts for more than 90% of all causes of RAS.^[16]^ Additionally, atherosclerotic disease is a chronic progressive disease; therefore, patients with high-grade ARAS experienced a process from low-grade stenosis to high-grade stenosis. The purpose of this study is to explore predictors of high-grade ARAS that may help clinicians timely recognize and manage high-grade ARAS in patients with CKD.

## 2. Methods

### 2.1. Study design

In this retrospective study, our research team collected clinical information on a total of 103 ARAS patients with CKD who were hospitalized in the Cardiovascular Department of the First Affiliated Hospital of Nanning Guangxi Medical University in China from January 2015 to December 2021. These patients all underwent renal arteriography for refractory hypertension, unexplained hypertension or the significant disparity between the size of their left and right kidneys. ARAS was detected by renal arteriography. The inclusion criteria include the presence of risk factors for atherosclerosis (diabetes, smoking, hyperlipidemia, age over 40, etc), imaging manifestations of atherosclerotic stenosis (conical stenosis or occlusion of the renal artery, eccentric stenosis with irregular plaque, intima calcification, mainly involving the proximal segment and opening of the renal artery, etc) detected by renal arteriography. The exclusion criteria include fibromuscular dysplasia (FMD), renal artery spasm Takayasu arteritis or other vasculitis, infectious diseases, cachexia, severe cardiopulmonary insufficiency, malignant tumors, poor general condition and contrast agent allergy. Then, patients were classified into 2 groups according to the stenotic extent of ARAS. A stenosis extent of the renal artery lumen greater than or equal to 50% was regarded as high-grade ARAS, and <50% was considered low-grade ARAS.^[4]^ CKD was diagnosed according to the KDIGO 2012 CKD Guideline.^[17]^

### 2.2. Laboratory measurements

We collected patients'medical records of their clinical characteristics and laboratory measurements. The clinical characteristics included age, gender, body mass index (BMI), CAD, hypertension, diabetes mellitus, smoking and drinking history. Hypertension was defined as systolic pressure ≥ 140 mm Hg and/or diastolic pressure ≥ 90 mm Hg or taking antihypertensive drugs.^[18]^ Drinking was defined as drinking more than 50 g/d of liquor (or other alcohol equivalent) for more than 1 year. Smoking was defined as smoking more than 1 cigarette per day for more than 1 year. Diabetes mellitus was defined as a FBG level ≥ 7 mmol/L, a nonFBG level ≥ 11.1 mmol/L, or the use of glucose-lowering medications.^[19]^ CAD was identified by clinical manifestations and coronary imaging or taking corresponding drugs (nitroglycerin, statins, antiplatelet drug, calcium channel blocker, etc). Estimated glomerular filtration rate (eGFR) was calculated using the chronic kidney disease epidemiology collaboration formula (CKD-EPI).^[20]^ The formulas for eGFR and BMI are as follows:

1. eGFR (mL/min/1.73 m^2^) = a(SCr/b)^c^(0.993)^age^, where *a* = 144 (female), 141 (male); *b* = 0.7 (female), 0.9 (male); when SCr ≤ 0.7 mg/dL, *c* = –0.329 (female), −0.411 (male); when SCr ≥ 0.7 mg/dL, *c* = –1.209 (female or male).
2. BMI (kg/m^2^) = weight (kg)/height^2^ (m^2^).

Routine laboratory biochemical parameters were collected at the same time and included lymphocyte and monocyte count, cystatin C, serum potassium, serum calcium, hemoglobin (Hb), fasting blood glucose (FBG), albumin (ALB), total cholesterol (TC), total triglyceride (TG), low-density lipoprotein (LDL), and high-density lipoprotein (HDL). eGFR was divided into 3 groups according to tertiles. The first tertile was <37.34 mL/min/1.73 m^2^, the second tertile was ≥37.34 mL/min/1.73 m^2^ and <66.33 mL/min/1.73 m^2^, and the third tertile was ≥66.33 mL/min/1.73 m^2^. Cystatin C was classified into 3 groups according to tertiles. The first tertile was <1.10 mg/L, the second tertile was ≥1.10 mg/L and <1.64 mg/L, and the third tertile was ≥1.64 mg/L. lymphocyte-to-monocyte ratio (LMR) was also divided into 3 groups according to tertiles. The first tertile was <2.31, the second tertile was ≥2.31 and <3.27, and the third tertile was ≥3.27. In addition, for study demand, LMR was further divided into 2 groups according to quantiles. The first quantile was <2.75, and the second quantile was ≥2.75.

To ensure the accuracy and uniformity of the results, all of the laboratory measurements were completed at the First Affiliated Hospital of Guangxi Medical University, Nannning, China.

### 2.3. Statistical analysis

All variables (age, gender, BMI, hypertension/CAD/DM/drinking/smoking history, FBG, TC, TG, LDL, HDL, SK, Sca, LMR, eGFR, cystatin C, Hb, ALB, lymphocyte, and monocyte count) were selected according to their potential clinical significance and findings from previous studies.^[21]^ All continuous variables were assessed for a normal distribution by the Kolmogorov–Smirnov test. Normally distributed variables are presented as the mean ± standard deviation, and variables with a skewed distribution are presented as the median with the interquartile range. Categorical variables are expressed as percentages. Normally distributed variables were further tested for homogeneity of variance. Independent-samples *T* tests were applied to compare normally distributed variables with homogeneity of variance between high-grade ARAS and low-grade ARAS groups, and the Wilcoxon test was used for variables with nonnormal distributions or nonhomogeneity of variance. The chi-square test was used to compare the constituent ratio of categorical variables between high-grade ARAS and low-grade ARAS) groups. Next, binary logistic regression analysis was used to explore independent risk factors. During binary logistic regression analysis, model I (crude model) and model II (adjusted for age, hypertension history, CAD history, smoking history, FBG, TC, and LDL) was established. Finally, the corresponding ROC curve was plotted, and the best threshold was calculated. An AUC of 0.5 to 0.6 was considered fail, 0.6 to 0.7 was general, 0.7 to 0.8 was good, 0.8 to 0.9 was excellent, and >0.9 was outstanding.^[22]^ Similarly, we have defined thresholds for sensitivity and specificity, with values > 70% considered clinically relevant. Data were analyzed using SPSS 26.0 (SPSS Inc., Chicago). GraphPad Prism 9 and the Xiantao Academy website were used for constructing images. A 2-tailed *P*-value < 0.05 was considered statistically significant.

## 3. Results

### 3.1. The baseline characteristics of ARAS in patients with CKD

The clinical characteristics of each group are listed in Table 1. The average age of the patients was 64.33 ± 11.25 years, and the average eGFR was 53.54 ± 29.04 ml/min/1.73 m^2^. Patients with high-grade ARAS were older than patients with low-grade ARAS. There were significant differences in smoking history, systolic pressure, diastolic pressure, eGFR, cystatin C, FBG, and LMR between the 2 groups. The levels of eGFR, FBG, and LMR were significantly lower in the high-grade ARAS group than in the low-grade ARAS group, while the levels of systolic pressure, diastolic pressure, cystatin C, and the proportion of smoking history were reversed.

**Table 1:** The baseline characteristics of renal artery stenosis in patients with CKD.

	All subjects (N = 103)	RAS < 50% (N = 37)	RAS ≥ 50% (N = 66)	*P*-value
Age (yr)	64.33±11.25	61.90 ±11.96	65.81 ±10.62	.087
Gender (female, %)	37 (35.9%)	16 (41%)	21 (32.8%)	.340
BMI (kg/m^2^)	24.77±3.89	25.17± 4.89	24.52 ±3.15	.418
Hypertension history (%)	96 (93.2%)	35 (89.7%)	61 (95.3%)	.635
DM history (%)	52 (50.5%)	22 (56.4%)	30 (46.9%)	.278
CAD history (%)	87 (84.5%)	30 (76.9%)	57 (89.1%)	.215
Smoking history (%)	37 (36.6%)	9 (23.7%)	28 (44.4%)	**.045** *
Drinking history (%)	34 (33.7%)	10 (26.3%)	24 (38.1%)	.257
Systolic pressure (mm Hg)	155.19 ± 29.09	147.51 ± 28.29	159.88 ± 28.80	**.036** *
Diastolic pressure (mm Hg)	84.94 ± 16.18	83.46 ± 16.24	85.84 ± 16.21	**.047** *
Lymphocyte (10^9^/L)	1.82±0.68	1.92 ±0.75	1.76 ±0.63	.251
Monocyte (10^9^/L)	0.62 (0.48, 0.77)	0.55 (0.46, 0.74)	0.68 (0.51, 0.84)	.058
Hb (g/L)	121.52±23.19	121.99± 19.79	121.24± 25.19	.874
ALB (g/L)	37.42±4.81	38.30± 4.51	36.86 ±4.94	.144
eGFR (mL/min/1.73m^2^)	53.54±29.04	65.18± 29.97	46.33 ±26.19	**.001** ***
Cystatin C (mg/L)	1.31 (0.98,1.99)	1.12 (0.86,1.44)	1.44 (1.11, 2.05)	**.007** **
FBG (mmol/L)	5.05 (4.49,6.34)	5.77 (4.63,8.37)	4.90 (4.37, 5.67)	**.011** *
TC (mmol/L)	4.64±1.31	4.67± 1.28	4.72 ±1.46	.412
TG (mmol/L)	1.46 (0.98,1.97)	1.36 (0.94,1.81)	1.46 (0.99,2.06)	.362
HDL (mmol/L)	1.04±0.28	1.01 ±0.28	1.06 ±0.28	.411
LDL (mmol/L)	2.74±0.98	2.64± 0.82	2.81± 1.06	.357
SK (mmol/L)	4.01±0.64	3.90± 0.58	4.07 ±0.67	.202
Sca (mmol/L)	2.22±0.16	2.23 ±0.15	2.22 ± 0.16	.801
LMR	2.75 (1.98,3.61)	3.23 (2.33,3.75)	2.56 (1.88,3.28)	**.016** *

The appropriated statistical description methods of variables were used.

ALB = albumin, BMI = body mass index, CAD = coronary artery disease, DM = diabetes mellitus, eGFR = estimated glomerular filtration rate, FBG = fasting blood glucose, Hb = hemoglobin, HDL = high-density lipoprotein, LDL = low-density lipoprotein, LMR = lymphocyte-to monocyte ratio, Sca = serum calcium, SK = serum potassium, TC = cholesterol, TG = triglycerides.

*< .05.

**< .01.

*** <.001.

### 3.2. The association between the above variables and high-grade ARAS in patients with CKD by binary logistic regression

To explore whether the above parameters with significant differences between the 2 groups were associated with high-grade ARAS, a binary logistic regression model was applied. The results are shown in Table 2. We found that only eGFR, cystatin C and LMR were still associated with high-grade ARAS in patients with CKD. In the crude model, which is referred to as model I, eGFR (OR = 0.976, 95% CI: 0.962–0.991, *P* = .002), cystatin C (OR = 1.981, 95% CI: 1.095–3.582, *P* = .024) and LMR (OR = 0.686, 95% CI: 0.487–0.966, *P* = .031) were significantly correlated with ARAS. After adjusting for potential confounding factors, such as age, hypertension history, CAD history, smoking history, FBG, TC and LDL, which is referred to as model II, eGFR (OR = 0.979, 95% CI: 0.962–0.996, *P* = **.017**), cystatin C (OR = 2.123, 95% CI: 1.118–4.030, *P* = **.021**) and LMR (OR = 0.639, 95% CI: 0.421–0.969, *P* = **.035**) were still correlated with high-grade ARAS.

**Table 2:** The results of association of parameters with renal artery stenosis by multivariate logistic regression.

	Model I OR (95% CI)	*P*-value	Model II OR (95% CI)	*P*-value
eGFR	0.976 (0.962, 0.991)	**.002** **	0.979 (0.962, 0.996)	**.017** *
Cystatin C	1.981 (1.095, 3.582)	**.024** *	2.123 (1.118, 4.030)	**.021** *
LMR	0.686 (0.487, 0.966)	**.031** *	0.639 (0.421, 0.969)	**.035** *

Model I: crude model; model II: adjusted for age, hypertension history, CAD history, smoking history, FBG, TC, and LDL.

CI = confidence interval, eGFR = estimated glomerular filtration rate, OR = odds ratio.

*< .05.

** <.01.

***<.001.

### 3.3. The constituent ratios of high-grade ARAS in eGFR, cystatin C and LMR tertiles

**Figure 1.**
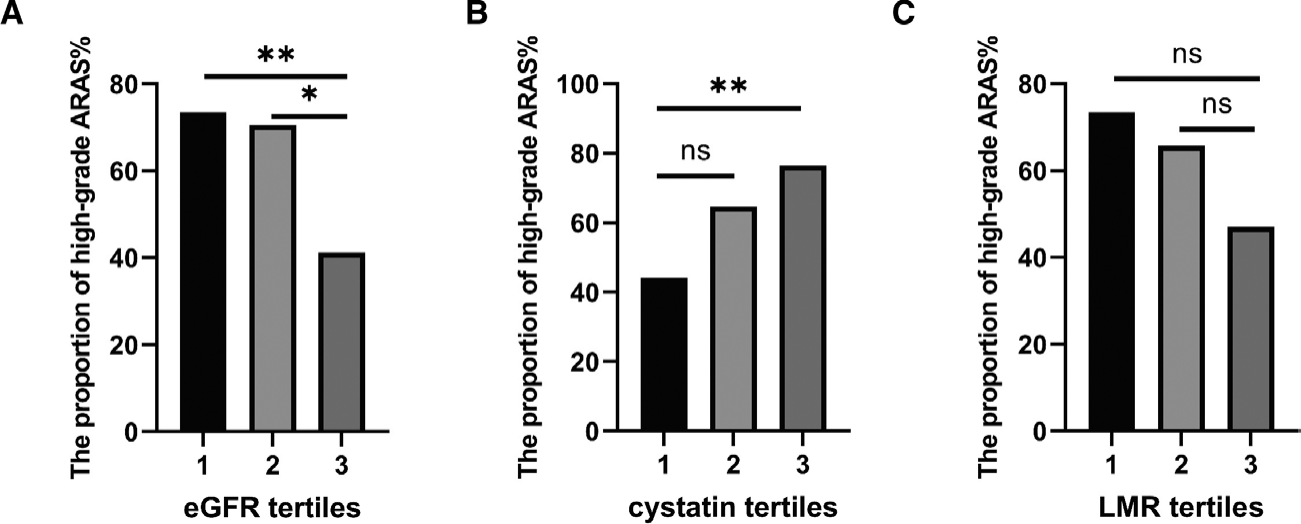
The proportion of high-grade atherosclerotic renal artery stenosis (ARAS) in eGFR tertiles, cystatin C tertiles, and lymphocyte-to-monocyte ratio (LMR) tertiles. (A) The proportion of high-grade ARAS in eGFR tertiles. (B) The proportion of high-grade ARAS in cystatin C tertiles. (C) The proportion of high-grade ARAS in LMR tertiles. Note: ^*^P < .05; ^**^P < .01; ^***^P < .001.

**Figure 2.**
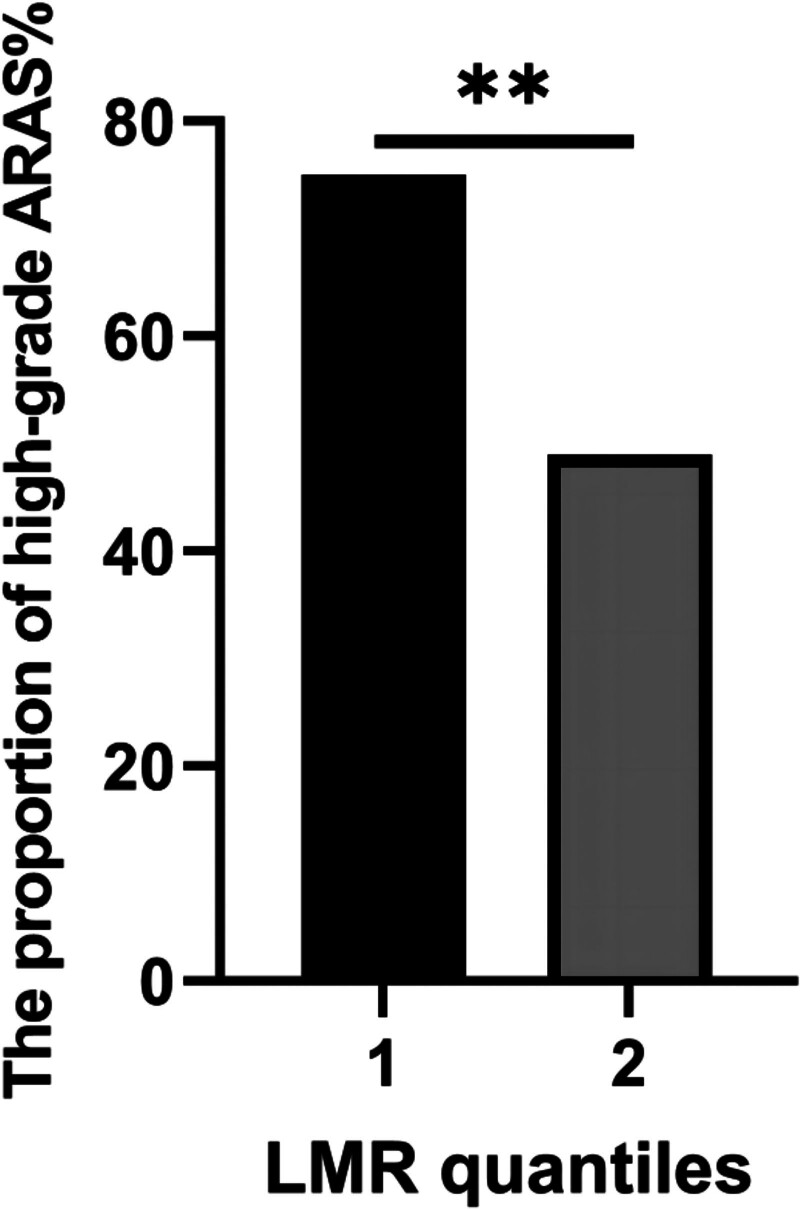
The proportion of high-grade ARAS in LMR quantiles. Note: ^*^P < .05; ^**^P < .010; ^***^P < .001. ARAS = atherosclerotic renal artery stenosis, LMR = lymphocyte-to-monocyte ratio.

Next, eGFR, cystatin C and LMR were divided into 3 groups according to their tertiles. The results of the proportion of high-grade ARAS in different tertiles are displayed in Figure 1. For eGFR, the chi-square test indicated that the proportion of high-grade ARAS in the third tertile (73.5% vs 41.2%, χ^2^ = 7.275, *P* = .007) and the second tertile (70.6 vs 41.2%, χ^2^ = 5.965, *P* = .015) was significantly higher than that in the first tertile (Fig. 1A). For cystatin C, the chi-square test showed that the proportion of high-grade ARAS in the third tertile was significantly higher than that in the first tertile (76.5% vs 44.1%, χ^2^ = 7.433, *P* = .006, Fig. 1B). For LMR, the chi-square test demonstrated that despite there being no significant difference among the 3 groups, the proportion of high-grade ARAS decreased with increasing tertile grade (Fig. 1C). Meaningfully, according to the quantiles of LMR, we found that the proportion of high-grade ARAS in the second quantiles of LMR was significantly lower than that in the first (49.0% vs 75.0%, χ^2^ = 7.387, *P* = .007), as shown in Figure 2.

### 3.4. The correlation between eGFR, cystatin C and LMR tertiles and high-grade ARAS in patients with CKD

**Figure 3.**
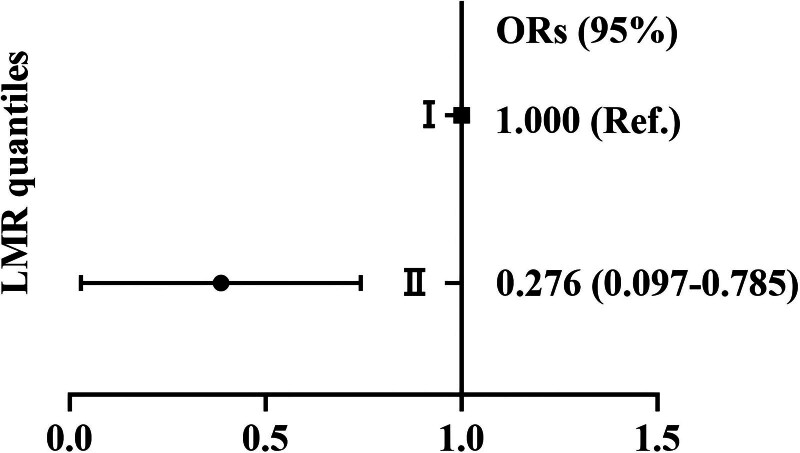
Association analysis of LMR quantiles with high-grade ARAS in patients with chronic kidney disease. ARAS = atherosclerotic renal artery stenosis, LMR = lymphocyte-to-monocyte ratio.

Next, we explored the association between eGFR, cystatin C and LMR tertiles and high-grade ARAS in patients with CKD. The results of the binary logistic regression are shown in Table 3. After correcting for age, hypertension history, CAD history, smoking history, FBG, TC and LDL, the risk for high-grade ARAS was decreased from the first tertile of eGFR to the third tertileof eGFR(OR = 0.261, 95% CI: 0.080–0.852, *P* = .026); the risk for high-grade ARAS was increased from the first tertile of cystatin C to third tertile of cystatin C (OR = 4.635, 95% CI: 1.345–15.975, *P* = **.015**). Among the tertiles of LMR, there were no significant differences. However, for the quantiles of LMR, the risk for high-grade ARAS was decreased from the first quantile of LMR to the second quantile of LMR (OR = 0.276, 95% CI: 0.097–0.785, *P* = .016) (Fig. 3).

**Table 3:** The association of high-grade RAS with eGFR, cystatin C, and LMR tertiles by multivariate regression analysis.

	Model I (OR 95% CI)	*P*-value	Model II (OR 95% CI)	*P*-value
eGFR (mL/min/1.73 m^2^)				
<37.34	1.000 (ref.)		1.000 (ref.)	
37.34 to 66.33	0.864 (0.299–2.495)	.787	0.793 (0.236–2.666)	.707
≥66.33	0.252 (0.091–0.701)	**.008** **	0.261 (0.080–0.852)	**.026** *
cystatin C (mg/L)				
<1.10	1.000 (ref.)		1.000 (ref.)	
1.10 to 1.64	2.322 (0.875–6.164)	.091	2.027 (0.645–6.369)	**.226**
≥1.64	4.117 (1.452–11.673)	**.008** **	4.635 (1.345–15.975)	**.015** *
LMR				
<2.31	1.000 (ref.)		1.000 (ref.)	
2.31 to 3.27	0.690 (0.246–1.939)	.482	0.727 (0.223–2.368)	.596
≥3.27	0.320 (0.116–0.885)	**.028** *	0.333 (0.090–1.227)	.098
LMR				
<2.75	1.000 (ref.)		1.000 (ref.)	
≥2.75	0.321 (0.139–0.738)	**.007** **	0.276 (0.097–0.785)	**.016** *

Model I: crude model; model II: adjusted for age, hypertension history, CAD history, smoking history, FBG, TC, and LDL. The first tertile as reference.

*< .05.

**< .01.

***<.001.

### 3.5. The predictive value of the above indicators for high-grade ARAS in patients with CKD

**Figure 4.**
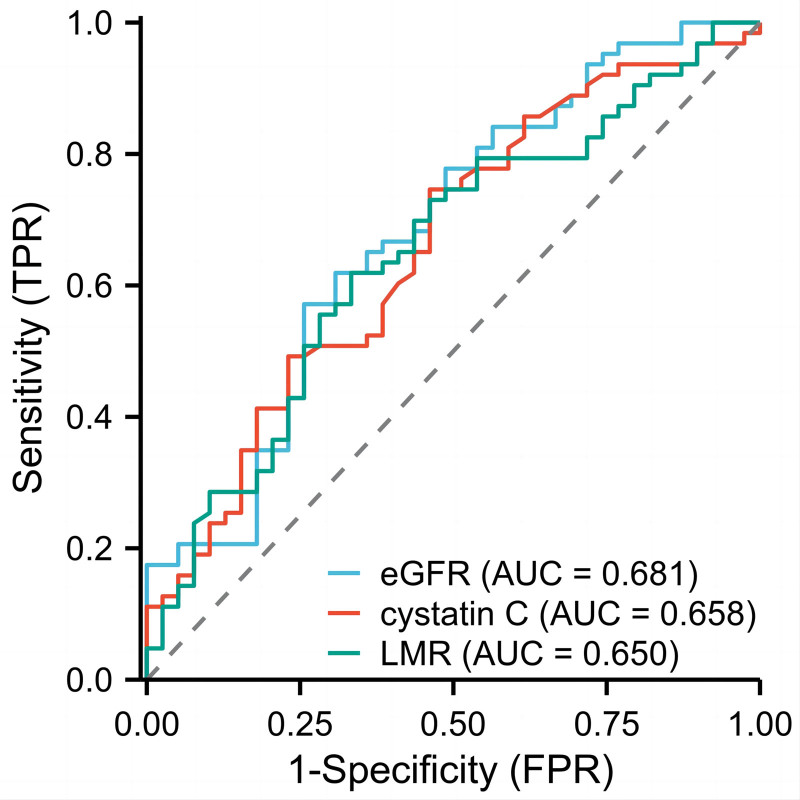
ROC curve of the diagnostic accuracy of eGFR, cystatin C and LMR for high-grade ARAS. ARAS = atherosclerotic renal artery stenosis, eGFR = estimated glomerular filtration rate, LMR = lymphocyte-to-monocyte ratio, ROC = receiver operating characteristic curve.

Finally, ROC analysis was used to determine the predictive value of the above variables for high-grade ARAS. The outcomes are shown in Figure 4 and Table 4. The AUC of eGFR was 0.681, with a sensitivity of 64.10% and specificity of 65.10%, and the AUC of cystatin C was 0.658, with a sensitivity of 74.60% and specificity of 53.85%. Furthermore, the AUC of LMR was 0.650, with a sensitivity of 66.70% and specificity of 62.00%.

**Table 4:** ROC analysis of eGFR, cystatin C, and LMR for predicting high-grade RAS in patients with CKD.

Variable	AUC	cutoff point	Sensitivity (%)	Specificity (%)	95% CI	*P*-value
eGFR (mL/min/1.73 m^2^)	0.681	55.45	64.10	65.10	0.572–0.789	.002**
cystatin C (mg/L)	0.658	1.13	74.60	53.85	0.549–0.768	.007**
LMR	0.650	2.77	66.70	62.00	0.540–0.760	.011*

AUC = the area under the curve, eGFR = estimated glomerular filtration rate, ROC = receiver operating characteristic curve.

*< .05.

** <.01.

***<.001.

## 4. Discussion

In this study, we reported that eGFR, cystatin C and LMR were associated with high-grade ARAS after correcting for confounding factors. eGFR and LMR, especially, also had relatively great prognostic value for high-grade ARAS in patients with CKD.

In line with our study, an Italian cohort study on predictive factors of ARAS in a large cohort of older CKD patients also established that eGFR is a significant predictors of ARAS in older CKD patients. In addition, patients with ARAS are usually accompanied by high prevalence of peripheral vessels atherosclerosis.^[23]^ It is well known that eGFR reflects renal function. At present, many studies have reported that the decline in renal function, that is, the decline in eGFR, is associated with atherosclerotic diseases, such as carotid artery stenosis, intracranial cerebral artery stenosis, coronary atherosclerotic diseases, and peripheral artery diseases.^[24–27]^ The decline in renal function can lead to inflammation, increased oxidative stress, damage to vascular endothelial cells, and disorders of calcium and phosphorus metabolism, and these factors promote the occurrence of atherosclerosis.^[28–30]^ However, the causal relationship between atherosclerosis and renal function is not clear. This is because renal function impairment may also be one of the stages of atherosclerosis development.^[31]^ Moreover, in this study, we found that hypertension, smoking, and drinking history accounted for a higher proportion in the high-grade stenosis group, and age was also higher, which were also risk factors for atherosclerosis. Furthermore, there was no clear correlation between the degree of RAS and renal function. Because eGFR is determined by renal blood flow and the hydrostatic pressure of glomerular capillaries, not all RAS will lead to renal ischemia and eGFR decline, regardless of the degree of stenosis.^[32]^

Numerous studies have reported that immune cells, such as neutrophils, lymphocytes, monocytes, and platelets, are important in the development of atherosclerotic vascular disease. As early as 1970, monocytes were reported to be involved in the development of atherosclerosis.^[33]^ Monocytes are involved in the entire process of atherosclerosis, including atherosclerotic plaque formation, acute inflammation, and repair stages.^[34]^ They contribute to atherogenesis by promoting leukocyte recruitment to plaques, leading to an inflammatory process.^[35]^ In addition, monocytes are the precursors of foam cells in atherosclerotic plaques. Initially, monocytes can differentiate into dendritic cells (DCs) and macrophages under different cytokines, such as granulocyte macrophage colony-stimulating factor or macrophage colony-stimulating factor.^[34,36]^ Then, macrophages ingest oxidized LDL via scavenger receptors, such as CD36, forming foam cells.^[37]^ DCs may also participate in the formation of foam cells.^[34]^ Furthermore, different monocyte subsets play different roles during the development of atherosclerosis, particularly those of the CD14^++^ subpopulation in humans and the Ly6C^hi^ subpopulation in mice, which are strongly associated with atherosclerosis.^[38]^ In contrast, lymphocytes exert a positive role in the development of atherosclerosis through their anti-inflammatory effects, and a low lymphocyte count has been shown to be correlated with atherosclerosis development.^[39,40]^ Recently, an article also reported that lymphocyte count was associated with low-grade RAS in female patients with CKD.^[21]^ The lymphocyte-to monocyte ratio (LMR) is a new type of inflammatory marker that can reflect the state of systemic inflammation. Compared with a single indicator, the combination of 2 indicators may reduce the impact of other unrelated factors on the test results and increase the stability and reliability of the results. It is easy to obtain and inexpensive. Therefore, it has been widely studied in recent years. For example, Gong et al^[41]^ and Yueqiao et al^[42]^ found that low LMR is independently and positively linked to coronary atherosclerosis. In addition, the LMR has been reported to be related to other diseases, such as cancer, infectious diseases and autoimmune disease.^[43–45]^ In the present study, we found that the LMR was related to high-grade ARAS in patients with CKD.

Cystatin C, which is a low-molecular-weight protein, can freely pass through the glomeruli and is almost completely reabsorbed by the proximal renal tubule and catabolized. It was less influenced by gender, age, inflammation, muscle mass and drugs.^[46]^ In the present study, we found that the level of serum cystatin C in the high-grade ARAS group was higher than that in the low-grade ARAS group. Therefore, this finding may imply that high-grade ARAS may further damage glomerular function compared to low-grade ARAS. Many studies have reported that cystatin C is associated with atherosclerosis. Cystatin C is a cysteine protease inhibitor. Therefore, it can regulate the production and degradation of the extracellular matrix in the vascular wall.^[47]^ Atherosclerosis occurs when the homeostasis between cystatin C and cysteine proteases is unbalanced.^[47,48]^ In addition, cystatin C is also a neutrophil growth factor that can affect the inflammatory status of plaques by affecting neutrophil biological properties, such as phagocytosis and migration.^[49]^ Hence, a higher level of cystatin C in patients with high-grade ARAS may indicate that the degree of atherosclerosis was more severe in the high-grade group than in the low-grade group.

This study has some limitations. First, this was a retrospective study; therefore, we could not conclude the causal relationship between eGFR, cystatin C and LMR and high-grade RAS in patients with CKD. Second, this was a single-center study, which is linked to the Asian geographical origin of the study population. Thus, the present results should be applied to other populations with caution. Third, the sample size of our study was small. Therefore, more conclusive data from large and well-designed longitudinal studies are needed to understand the causal relationship between eGFR, cystatin C and LMR and high-grade ARAS in patients with CKD.

## 5. Conclusions

Taken together, our study provided evidence that eGFR, cystatin C and LMR were associated with high-grade ARAS in patients with CKD as well as predictive parameters of high-grade ARAS. These predictors, especially eGFR and LMR, may help clinicians recognize high-grade RAS in patients with CKD in the future.

## Acknowledgments

We are grateful to Xiantao Academy website for its drawing function(www.xiantaozi.com).

## Author contributions

**Conceptualization:** Jun Ouyang.

**Data curation:** Jun Ouyang, Kequan Chen.

**Formal analysis:** Jun Ouyang.

**Funding acquisition:** Jiangnan Huang.

**Writing – original draft:** Jun Ouyang.

**Writing – review & editing:** Hui Wang, Jiangnan Huang.
